# *SOX15* Contributes to the Maintenance of Pluripotency in Porcine Embryonic Stem Cells

**DOI:** 10.3390/cells15141283

**Published:** 2026-07-17

**Authors:** Chenghe Jian, Yanjiao Lv, Miao Xu, Hongxing Wang, Han Du, Yiyang Wang, Muhan Su, Jun Song, Tingsheng Yan, Zhonghua Liu

**Affiliations:** 1 College of Life Science, Northeast Agricultural University, Harbin 150030, China; jianchenghe@neau.edu.cn (C.J.); wanghongxing@neau.edu.cn (H.W.); duhan@neau.edu.cn (H.D.); wangyiyang@neau.edu.cn (Y.W.); sumuhan@neau.edu.cn (M.S.); songjun@neau.edu.cn (J.S.); 2 Key Laboratory of Animal Cellular and Genetics Engineering of Heilongjiang Province, Northeast Agricultural University, No. 59 Mucai Street, Harbin 150030, China; lvyanjiao2026@163.com (Y.L.); xumiao0615@163.com (M.X.)

**Keywords:** pig, embryo-derived stem cells, *SOX15*, transcription factor pluripotency

## Abstract

**Highlights:**

**What are the main findings?**

- *SOX15* exhibits a unique spatiotemporal expression pattern in pigs, being robustly upregulated from the 2-cell stage and remaining highly expressed in the epiblast, which contrasts sharply with its low expression in mouse embryos.
- Stable *SOX15* knockdown is associated with impaired pluripotency-associated features, including reduced colony integrity, diminished alkaline phosphatase activity, and downregulation of core factors (*OCT4*/*NANOG*). Additionally, loss of *SOX15* compromised embryoid body formation and abolished teratoma-forming capacity.

**What are the implications of the main findings?**

- Challenges the rodent-centric paradigm of pluripotency, suggesting that *SOX15* may act as a lineage-adapted regulator with increased functional weight in pigs that has acquired critical functional weight in large mammals like pigs.
- Provides a conceptual framework for optimizing livestock stem cell technologies, and identifying *SOX15* serves as a species-specific regulator that plays an important role in establishing stable naive pluripotent stem cell lines in pigs.

**Abstract:**

Understanding how pluripotency regulatory networks evolve across mammals remains a central question in developmental and stem cell biology. While rodent models have defined the canonical core circuitry of pluripotency, the extent to which these regulatory hierarchies are conserved in large mammals is unclear. Here, we provide evidence that *SOX15* contributes to the species-specific regulatory network as a modulator that sustains pluripotency and preserves functional differentiation capacity in porcine embryo-derived stem cells. Comparative sequence analysis revealed strong conservation of the *SOX15* HMG domain across mammals, yet promoter divergence suggested lineage-specific regulatory evolution. Transcriptomic profiling demonstrated that, unlike in mice, *SOX15* is robustly upregulated from the 2-cell stage and remains highly expressed in the porcine epiblast, coinciding with key windows of pluripotency establishment. In porcine embryo-derived stem cells, stable *SOX15* knockdown resulted in reduced colony integrity, diminished alkaline phosphatase activity, impaired proliferation, and downregulation of core pluripotency genes, including *OCT4* and *NANOG*. Furthermore, loss of *SOX15* disrupts embryoid body formation and abolishes teratoma-forming capacity in vivo. This deficit likely reflects impaired pluripotency, but may also be attributed to compromised cell survival or proliferative fitness following transplantation. Notably, comparable perturbations in mouse models do not produce equivalent phenotypes, underscoring a lineage-dependent functional divergence. Together, our findings suggest that *SOX15* contributes to the support of the porcine pluripotency network, and hint at evolutionary plasticity in the hierarchical architecture of mammalian pluripotency. These findings move beyond the rodent-centric paradigm and offer a refined perspective on the evolutionary plasticity of pluripotency networks, highlighting *SOX15* as a pivotal lineage-specialized node governing naive pluripotency in large mammals. Notably, these inferences are based on functional perturbation using a single validated miRNAi construct; definitive confirmation of *SOX15*-specific causality will require future orthogonal validation via independent knockdown sequences, CRISPR interference, or RNAi-resistant rescue experiments.

## 1. Introduction

In mammalian early embryogenesis and pluripotency regulatory networks, members of the SOX transcription factor family occupy central positions [1,2,3]. Among them, *SOX2* is widely recognized as an indispensable core factor for maintaining self-renewal and regulating cell fate determination. However, *SOX2* does not function in isolation; rather, it anchors a highly interconnected pluripotency network. Mechanistically, *SOX2* cooperates with *OCT4* to activate downstream target genes through the canonical SOX-OCT composite motif, forming a foundational paradigm of pluripotency regulation [4]. This core circuitry is essentially supported by other fundamental regulators, such as KLF4, which stabilizes feed-forward transcriptional loops, and *c-MYC*, a central hub coordinating the specific metabolic and cell-cycle landscapes required for the pluripotent state [5,6,7,8]. Furthermore, integration with surface receptors, such as the receptor tyrosine kinase c-Kit [9], provides necessary extracellular signaling cues to support progenitor cell survival [10]. Within this complex SOX-anchored regulatory ecosystem. *SOX15*, a member of the SOX family Group G, shares high sequence homology with *SOX*2 in the conserved HMG-box DNA-binding domain [11]. Due to this similarity and its expression in undifferentiated mouse ESCs (mESCs) [12,13], *Sox15* was initially regarded as a potential auxiliary or redundant factor for *Sox2*. Indeed, *Sox15* can bind *Oct4* and partially compensate for *Sox2* deficiency in vitro [4,13]. However, genetic studies in mice revealed that *Sox15* knockout animals develop normally and remain fertile, although they do exhibit only subtle differentiation delays in specific cell lineages [12,14]. These findings have led to the prevailing view that *SOX15* plays a relatively minor or redundant role in mammalian pluripotency regulation.

Importantly, this conclusion is largely derived from mouse-centric studies [13]. Whether such an interpretation universally applies to other mammals remains unclear [12]. Increasing evidence suggests that pluripotency regulatory networks differ substantially between rodents and large mammals, particularly in species such as pigs, whose embryonic development, epiblast formation, and placental structures exhibit significant divergence from those of mice [15]. Moreover, the establishment and maintenance of porcine pluripotent stem cells (pPSCs) remain technically challenging, implying that the regulatory circuitry governing pluripotency in pigs may rely on distinct or species-specific core factors. As a mammal-specific gene that emerged relatively late during evolution [12], *SOX15* may have undergone functional specialization in different mammalian lineages. However, its dynamic expression pattern during porcine early embryogenesis and its functional role in porcine embryo-derived stem cells have not been systematically investigated. This knowledge gap limits our understanding of porcine pluripotency networks and constrains efforts to establish stable naïve pluripotent stem cell lines with full developmental potential in pigs [16].

Given the increasing importance of pigs as biomedical models and agricultural resources, elucidating species-specific regulators of pluripotency in this major livestock species is both biologically and translationally significant [16]. However, a major gap remains in understanding how these species-specific factors mechanistically interface with the canonical core circuitry. Specifically, whether *SOX15* acts through canonical SOX-OCT composite motifs or physically interacts with core factors (e.g., *SOX2*, *OCT4*) to orchestrate pluripotency in large mammals remains unknown.

To improve the robustness of our findings and minimize potential cell line- and culture system-dependent biases, we employed two previously established porcine pluripotent stem cell models, pLCDM and pEPSC, which differ in developmental origin and culture conditions. pLCDM cells, derived from parthenogenetic embryos, and pEPSCs, derived from in vivo fertilized embryos, represent different developmental ontogenies and are maintained under vastly different culture conditions. By assessing *SOX15* function in these orthogonal systems, we aim to provide comprehensive evidence that its role in maintaining pluripotency is a generalized phenomenon, independent of specific culture microenvironments or epigenetic backgrounds. We sought to move beyond the rodent-centered paradigm and systematically investigate the expression characteristics and functional role of *SOX15* in porcine early embryos and embryo-derived stem cells. Through cross-species sequence comparison and developmental transcriptomic analyses, we identified a distinct and sustained high-expression pattern of *SOX15* in porcine early embryos, particularly during the epiblast stage, in contrast to its low expression in mice. Furthermore, using stable *SOX15* knockdown models in porcine embryo-derived stem cells, we provide direct functional evidence that *SOX15* is required for maintaining several pluripotency-associated features. Our findings suggest a potential species-specific regulatory role of *SOX15* in porcine pluripotency and provide new molecular insights into the evolution and diversification of mammalian pluripotency networks.

## 2. Materials and Methods

### 2.1. Electrical Activation of Oocytes

Denuded oocytes with 1st PB were selected and transferred into a chamber with 2 electrodes spaced 3.2 mm apart and filled with activation solution (0.28 M mannitol, 0.1 mM CaCl_2_, 0.1 mM MgSO_4_, and 0.5 mM HEPES) and activated by electric stimulation with a single direct current pulse of 1.5 kV/cm for 60 μs using a BXT Electro-Cell Manipulator 2001 (BXT Inc., San Diego, CA, USA). Activated oocytes were washed and transferred into wells containing 500 μL of porcine zygote medium-3 (PZM-3), and cultured under a humidified atmosphere of 5% CO_2_, 5% O_2_, and 90% N_2_ at 38.5 °C for 7 days.

### 2.2. Preparation of Feeder Cells

Pregnant C57BL/6 mice at gestational day 13.5 were obtained from Harbin Medical University and euthanized by cervical dislocation. Embryos were collected and rinsed in physiological saline supplemented with double-strength penicillin–streptomycin (CELLGRO, Cat. No. 30002 CI). Dorsal tissues were isolated, minced thoroughly, and digested with 1.6 mg/mL DNase I (GIGMAD, Cat. No. 426). Digestion was terminated by adding medium containing 20% fetal bovine serum (FBS; BIOIND, Cat. No. 040011A Israel). After centrifugation, the cell pellet was resuspended and seeded into 90 mm culture dishes. Cells were maintained at 37 °C in a humidified atmosphere containing 5% CO_2_.

To prepare feeder cells, mouse embryonic fibroblasts (MEFs) were treated with 200 µL of 10 µg/mL mitomycin C (Sigma, Cat. No. M4287 USA) per 90 mm dish for 2.5 h. After treatment, cells were washed three times with PBS and dissociated with 0.25% trypsin (Gibco, Cat. No. 25300062 USA) for 2 min. Trypsinization was stopped by adding medium containing 15% FBS. The cell suspension was centrifuged at 1500 rpm for 3 min. The supernatant was discarded, and the cell pellet was resuspended in freezing medium and stored at −80 °C.

### 2.3. Source of Pig Embryo-Derived Stem Cell Lines

The porcine LCDM (pLCDM) stem cell line, which is parthenogenetic in origin, was previously established and characterized by our group [17]. For the porcine expanded pluripotent stem cells (pEPSCs), we utilized a well-characterized cell line derived in vivo. These cells were derived under conditions involving selective inhibition of the MAPK pathway, which has been demonstrated to sustain pluripotency more effectively than conventional methods [18]. In the present study, all cell cultures were routinely tested and confirmed to be free of mycoplasma contamination prior to experimentation.

### 2.4. Culture of pLCDM and pEPSC

pLCDM and pEPSC cells were cultured on mitomycin C-inactivated mouse embryonic fibroblast (MEF) feeder layers. The medium for both cell lines was refreshed daily. To maintain the cells in an undifferentiated state, the following culture protocol was strictly implemented: cells were passaged every 3 days at a ratio of 1:3 to 1:5 using 0.05% trypsin (Gibco, Cat. No. 25300062 USA). Following passaging, cells were seeded in medium supplemented with 10 μM Y27632. The specific compositions of the pLCDM and pEPSC culture systems are detailed below.

The composition of the pLCDM medium was as follows: 24 mL DMEM/F12 (Gibco, Cat. No. 1330-032 USA), 24 mL Neurobasal (Gibco, Cat. No. 21103-049 USA), 500 μL double antibody (CELLGRO, Cat. No. 30-002-CI USA), 500 μL L-glutamine (Glu; Gibco, Cat. No. 17504-044 USA), 500 μL non-essential amino acids (NEAA; Invitrogen, Cat. No. 11140-050 USA), 500 μL B27 supplement (Gibco, Cat. No. 17504-044 USA), 250 μL N2 supplement (Gibco, Cat. No. 17502-048 USA), 100 μL β-mercaptoethanol (β-Me; Gibco, Cat. No. 21985-023 USA), 156.25 μL 2% bovine serum albumin (BSA; Sigma, Cat. No. A3311 USA), 10 ng/mL human leukemia inhibitory factor (hLIF; Millipore, Cat. No. LIF1010 USA), 3 μM CHIR 99021 (Stemgent, Cat. No. 04-0004 USA), 2 μM (S)- (+)-Dimethindene maleate (TOCRIS, Cat. No. 1425 UK), and 2 μM Minocycline hydrochloride (Abcam, Cat. No. ab120661 UK). During cell recovery or passaging, Y27632 (EMD 4Biosciences, Cat. No. 688000 USA) was added to the medium to a final concentration of 10 μM.

Cells were maintained in a fully humidified incubator at 39 °C under an atmosphere of 5% O_2_, 5% CO_2_, and 90% N_2_.

The modified pEPSC culture medium was prepared according to our previously established protocol [18]: 47.4 mL DMEM/F12, 500 μL double antibody, 500 μL L-glutamine (Glu), 500 μL non-essential amino acids (NEAA), 500 μL B27 supplement, 250 μL N2 supplement, 100 μL β-mercaptoethanol (β-Me), 150 μL fetal bovine serum (FBS), 3250 μg Vitamin C (Sigma, Cat. No. A5960 USA), 10 ng/mL human leukemia inhibitory factor (hLIF), 20 ng/mL Activin A (R&D Systems, Cat. No. 338-AC-10 USA), 2.5 μM XAV939 (Sigma, Cat. No. X3004 USA), 0.3 μM WH (TOCRIS, Cat. No. 5431 UK), 0.2 μM CHIR99021, 0.25 μM SB590885 (R&D Systems, Cat. No. 2650 USA), and 4 μM JNK Inhibitor VIII (TOCRIS, Cat. No. 3222 UK). Cells were maintained in a fully humidified incubator at 39 °C under an atmosphere of 5% O_2_, 5% CO_2_, and 90% N_2_.

Both pLCDM and pEPSC cell lines were selected based on their established genomic stability and high pluripotency marker expression. The inclusion of these two independent models allows for cross-validation of *SOX15* function, reducing the likelihood of the possibility that the observed phenotypes are artifacts of a specific culture system or the result of parthenogenetic origin [17,18,19].

### 2.5. Culture of Mouse EPS Cells

MEPSCs were derived from C57BL/6 or C57BL/6 × 129 F1 blastocysts and plated onto mitotically inactivated mouse embryonic fibroblast MEF feeders in N2B27-LCDM medium. The resulting cell line was designated mLCDM-EPSCs. Colonies typically formed within four days and were mechanically dissociated into small clumps for passaging. Cells were subcultured every 2–4 days via single-cell dissociation using 0.05% trypsin-EDTA at a split ratio of 1:3 to 1:10.

### 2.6. Sequencing Samples and High-Throughput Data Sources

The sequencing data utilized in this report were sourced from public repositories. For porcine oocytes, 2-cell, 4-cell, 8-cell, and ICM stages, data were obtained from GSE139512 and GSE112380, respectively. For mouse 1-cell, 2-cell, 4-cell, 8-cell, ICM, and epiblast stages, data were acquired from GSE71434 and GSE273917, respectively. Raw sequencing reads were first subjected to quality control using Fast QC (v0.12.1). Adapter sequences and low-quality bases were removed using Trim Galore (v0.6.10) with default parameters. The remaining high-quality clean reads were then aligned to their respective reference genomes using STAR (v2.7.10b). Specifically, mouse reads were mapped to the Mus musculus reference genome (GRCm39) with GENCODE (vM38) gene annotation, while pig reads were mapped to the Sus scrofa reference genome (Sscrofa11.1) with NCBI RefSeq (GCF_000003025.6) gene annotation. Subsequent processing of alignment files, including sorting and indexing, was performed using SAMtools (v1.16.1). Gene-level quantification was carried out using featureCounts from the Subread package (v2.0.6) to generate raw read counts. The resulting raw count matrix was subsequently imported into R software (R 4.4.3). To account for variations in gene length and sequencing depth, Fragments Per Kilobase of transcript per Million mapped reads (FPKM) values were calculated using custom R scripts based on the effective gene lengths derived from the respective annotation files.

### 2.7. Vector Construction

Total RNA was extracted from the porcine SOX15-high-expression cell line pEPSC and reverse-transcribed to obtain cDNA. The porcine *SOX15* cDNA fragment (coding sequence length: 702 bp) was amplified by PCR using primers (Supplementary Table S1) designed with an AgeI restriction site (upstream) and an EcoRI site (downstream). The PCR product was then ligated into the pMACS vector using T4 DNA Ligase. An HA tag was subsequently inserted in-frame upstream of the SOX15 coding sequence within the pMACS vector, resulting in the final *SOX15* overexpression plasmid carrying an N-terminal HA tag.

The miRNAi sequences targeting porcine *SOX15* were designed using the BLOCK-iT™ RNAi Designer tool. The specific oligonucleotide sequences (Osi-F and Osi-R) are listed in Supplementary Table S1. These oligonucleotides were synthesized by Guangzhou IGE Biotechnology Ltd.

The annealed oligonucleotides were cloned into the linearized pcDNA™ 6.2-GW/EmGFP vector according to the manufacturer’s instructions of the BLOCK-iT™ Pol II miR RNAi Expression Vector Kit (Thermo Fisher Scientific, Cat. No. K493600 USA), generating the entry plasmid pcDNA™ 6.2-GW/EmGFP-miR-SOX15.

The miRNA expression cassette (containing the EmGFP reporter and the *SOX15*-specific miRNAi sequence) was amplified from the pcDNA™ 6.2-GW/EmGFP-miR-*SOX15* plasmid by PCR using primers (Supplementary Table S1) containing XbaI (upstream) and EcoRI (downstream) restriction sites. The resulting PCR fragment and the FUW lentiviral backbone were digested with XbaI and EcoRI. The purified fragment was then ligated into the digested FUW vector, yielding the final lentiviral expression plasmids FUW-EmGFP-*SOX15*miR (for *SOX15* knockdown) and the control vector FUW-EmGFP.

### 2.8. Lentivirus Packaging

293T cells were seeded in culture dishes pre-coated with 50 mg/mL poly-D-lysine (PDL; Sigma, Cat. No. P6407-5MG USA) and cultured until they reached 70–90% confluency. Viral packaging plasmids pMDLg, pRSV-Rev, pMD2G and the target plasmid were mixed thoroughly in 1 mL Opti-MEM (Gibco, Cat. No. 31985-070 USA). Polyethylenimine 4000 (PEI 4000) was added immediately, and the mixture was vortexed for 10 s and incubated at room temperature for 15 min. The culture medium of 293T cells was replaced with serum-free Opti-MEM, and the plasmid–PEI complex was added to the cells. After 8 h of transfection, the medium was replaced with complete medium containing 2% fetal bovine serum (FBS).

Viral supernatants were collected at 24 h and 48 h after medium replacement, centrifuged at 3000× *g* for 10 min at 4 °C, mixed with PEG, and incubated at 4 °C overnight. The mixture was further centrifuged at 4000× *g* for 30 min at 4 °C. The supernatant was discarded, and the viral pellet was resuspended in lentivirus resuspension buffer. The concentrated viral suspension was subjected to viral titer determination and stored at −80 °C until use.

### 2.9. Lentiviral Infection of Porcine Embryo-Derived Stem Cells

For lentiviral infection, the regular culture medium of porcine embryo-derived stem cells was replaced with antibiotic-free medium before infection. The prepared lentiviral suspension was directly added to the antibiotic-free medium. After 24 h of infection, the medium was replaced with regular culture medium. GFP expression was observed under a fluorescence microscope at 48 h post-infection to verify infection efficiency.

### 2.10. Alkaline Phosphatase Staining and Quantification

Cells were fixed with 4% (*w*/*v*) paraformaldehyde for 90 s at room temperature and then washed three times with DPBS. Alkaline phosphatase activity was determined using the BCIP/NBT Alkaline Phosphatase Color Development Kit (Beyotime, Cat. No. C3206 China). Stained cultures were observed under a light microscope. A colony was strictly defined as AP-positive only if it met the following criteria: exhibiting a uniform dark purple/blue-black coloration, showing specific cytoplasmic staining, and containing less than 30% unstained area within the colony. To quantify the AP-positive rate, at least five random fields per well were selected, with approximately 50–100 colonies evaluated per field. The AP-positive colony rate was calculated as follows: AP-positive colony rate (%) = (Number of AP-positive colonies/Total number of colonies) × 100%. All experiments were performed using at least three independent biological replicates, and data are presented as mean ± standard deviation (SD).

### 2.11. Immunofluorescence Staining

Cells were fixed with 4% paraformaldehyde at room temperature for 30 min. Following fixation, cells were permeabilized with 1% Triton X-100 in PBS for 1 h at 37 °C, and then blocked with blocking solution (PBS containing 0.1% Tween-20, 0.01% Triton X-100, and 1% BSA) for 1 h at 37 °C.

Primary antibodies were incubated with the cells at 4 °C overnight (1:50): Anti-NANOG (PEPROTECH, Cat. No. 500-p236 USA), Anti-SOX2 (Santa Cruz, Cat. No. sc-17320 USA), Anti-OCT4 (Santa Cruz, Cat. No. sc-8628 USA), Anti-SOX15 (Thermo Fisher Scientific, Cat. No. PA5-720115), Anti-GATA6 (Abcam, Cat. No. ab22600 UK), Anti-α-SMA (Abcam, Cat. No. ab5694 UK), and Anti-β-TUBULIN (Abcam, Cat. No. ab6046 UK). After incubation, cells were washed three times with washing buffer for 5 min each.

Secondary antibodies (1:200) (donkey anti-goat IgG 488, Invitrogen, Cat. No. A11055; donkey anti-rabbit IgG 488, Invitrogen, Cat. No. A21206 USA) were added and incubated at 37 °C for 1 h, followed by three washes with washing buffer for 5 min each. Cell nuclei were counterstained with Hoechst 33342 for 10 min. Finally, cells were mounted and observed under a fluorescence microscope.

Immunofluorescence images were acquired using identical exposure time, gain, and illumination settings for all groups within the same experiment. Quantitative analysis of fluorescence intensity was performed using ImageJ software (ImageJ 1.8.0.345). Regions of interest (ROIs) were delineated based on Hoechst-positive nuclei or colony boundaries, and the mean fluorescence intensity was measured after background subtraction. Each experimental condition included at least three independent biological replicates. For each biological replicate, at least five random fields were captured, and 30–50 cells or colonies were analyzed per field. Data were presented as mean ± standard deviation (SD).

### 2.12. Detection and Analysis of EB Formation and Spontaneous Differentiation

Cells were dissociated with 0.05% trypsin (39 °C, 3 min), centrifuged at 1000 rpm for 3 min, and seeded into dishes at 4 × 10^5^ cells/dish in 15% FBS medium for suspension culture. On the second day, half of the medium was replaced. Embryoid body (EB) formation was observed after 4–7 days, and images of morphologically regular EBs were captured to measure EB diameter. The EBs were harvested to analyze the expression of three germ layer markers. Subsequently, the EBs were transferred onto 24-well plates pre-coated with collagen (pre-incubated for 30 min) using an oral pipette. Cell growth was monitored on day 7 post-attachment, and the expression of three germ layer marker genes was assessed by immunofluorescence.

### 2.13. RNA Extraction and Real-Time Fluorescence Quantitative PCR

Total RNA was extracted using the PureLink-RNA small kit (Invitrogen, Cat. No. 12183018A USA); reverse transcription was performed using the High-Capacity cDNA reverse transcription kit (ABI, Cat. No. 4368814 USA): The reaction protocol: a. 25 °C for 10 min; b. 37 °C for 120 min; c. 85 °C for 5 s; d. 4 °C for 10 min.

Real-time quantitative PCR was carried out using the TB Green^®^ Premix Ex Taq™ (Tli RNaseH Plus) real-time quantitative PCR kit (Takara, Cat. No. DRR066A Japan). The PCR primers used are detailed in Table S2.

### 2.14. Cell Doubling Time Detection

Cells were cultured in 24-well plates. One group of cells was digested every 12 h, and the cells were resuspended in 1 mL DPBS solution. Then, 10 µL of the cell suspension was dropped onto the counting plate and the cell number was calculated using a cell counter. The formula for calculating the doubling time is: Doubling time (DT) = t × [lg2/(lgNt − lgN0)], where t is the cell culture time (h); Nt is the cell number recorded t h after inoculation; N0 is the cell number recorded t h after inoculation.

### 2.15. Phylogenetic Analysis

SOX15 protein sequences from various species were retrieved from the NCBI database. The identity of each sequence was confirmed based on gene annotation and conserved domain analysis, and all sequences were verified to contain the characteristic HMG-box domain. Multiple sequence alignment was performed using MAFFT with default parameters, followed by manual inspection and trimming of poorly aligned regions when necessary. A phylogenetic tree was then constructed based on the optimized alignment using the maximum-likelihood method implemented in IQ-TREE. The best-fit amino acid substitution model was selected automatically by Model Finder. Branch support was evaluated using 1000 ultrafast bootstrap replicates and 1000 SH-aLRT tests. The resulting tree was visualized using iTOL, and midpoint rooting was applied.

### 2.16. Data Statistical Analysis

Statistical analysis was performed using SPSS software (IBM SPSS Statistics 29.0). GraphPad Prism 9 was used exclusively for generating graphical visualizations.

Prior to conducting parametric tests, data were assessed for normality using the Shapiro–Wilk test and for homogeneity of variances using Levene’s test. For comparisons between two groups, an unpaired two-tailed Student’s *t*-test was used. For comparisons among three or more groups, one-way analysis of variance (ANOVA) was performed. When significant differences were detected by one-way ANOVA, Dunnett’s post hoc test was used to compare each experimental group with the corresponding control group. Data are presented as the mean ± standard deviation (SD). A value of *p* < 0.05 was considered statistically significant. * represents *p* < 0.05, ** represents *p* < 0.01, and *** represents *p* < 0.001.

## 3. Results

### 3.1. Sequence Characteristics and Evolutionary Conservation Analysis of the SOX15 Gene

To elucidate the cross-species conservation and structural characteristics of *SOX15*, we first performed a systematic analysis of the porcine *SOX15* gene sequence. The results showed that the porcine *SOX15* gene is located on chromosome 12, with a coding sequence (CDS) of 702 bp. The porcine SOX15 protein consists of 234 amino acids and is encoded by two exons: exon 1 is 533 bp in length, and the known region of exon 2 is 169 bp (Supplementary Figure S1A).

All members of the SOX family are characterized by a conserved high-mobility group (HMG) domain, which is crucial for DNA-binding activity. In porcine *SOX15*, the HMG domain localizes to amino acid residues 59–117 (Supplementary Figure S1B).

*SOX2*, a member of the *SOXB1* subgroup, is located on porcine chromosome 13. It has a 960 bp CDS encoding a 320-amino-acid protein with a single-exon structure. *SOX2* plays pivotal roles in early embryonic development and cell fate determination. Notably, *SOX2* is co-expressed at high levels with *SOX15* (a member of the SOXG subgroup) in undifferentiated stem cells, and their sequences share high similarity. We therefore compared the HMG domain sequences of *SOX2* and *SOX15* across different species to explore their evolutionary relationship (Supplementary Figure S1D).

To further investigate the cross-species conservation of *SOX15*, we compared the position and length of the *SOX15* HMG domain among mice, humans, pigs, cattle, and goats. The results demonstrated that the HMG domain length is highly similar across these species, and its relative position within the amino acid sequence is also remarkably conserved (Supplementary Figure S1C), indicating that these sequences are evolutionarily conserved and exert important biological functions.

**Figure 1:**
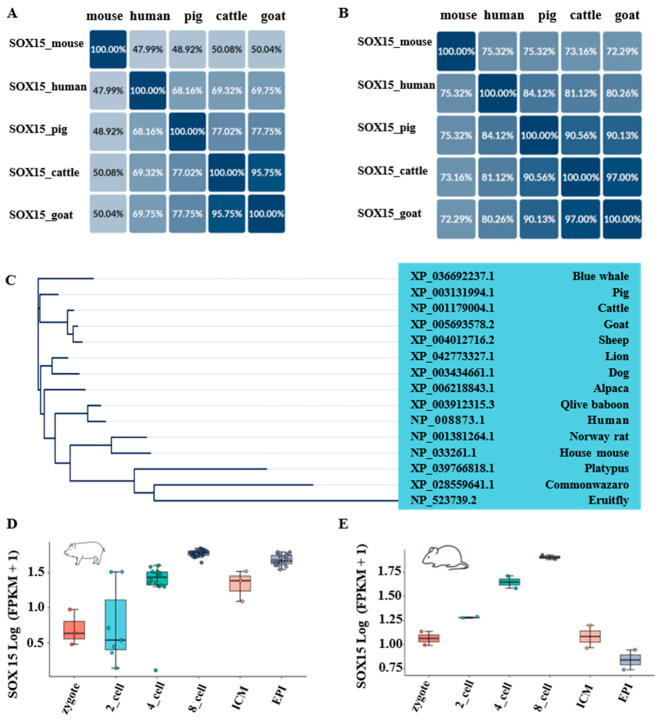
Analysis of the sequence characteristics and evolutionary conservation of the *SOX15* gene. (**A**) Comparative analysis of SOX15 promoter region sequences across multiple species; (**B**) multiple sequence alignment of SOX15 amino acid sequences from various species; (**C**) *SOX1*5 multispecies phylogenetic tree; (**D**) expression of *SOX15* at various stages of early pig embryonic development; (**E**) expression of S*ox15* at various stages of early mouse embryonic development.

To verify the conservation of *SOX15* across multiple mammalian species, we aligned the promoter region sequences of *SOX15* (ranging from 2000 bp upstream of the transcription start site to the translation initiation codon ATG) from the aforementioned mammals. Sequence comparison revealed that the similarity between pigs and other large mammals (cattle and goats) exceeded 60%, whereas the similarity between pigs and mice was only 48.92%. Sequence comparison showed that the upstream region of porcine *SOX15* shared higher pairwise similarity with cattle and goat than with mouse. (Figure 1A). However, because pairwise promoter similarity alone is insufficient to infer conserved transcriptional regulation, these results should be interpreted as evidence of sequence-level divergence in *SOX15* upstream regions among mammals, rather than direct evidence for conserved regulatory mechanisms. Further analyses of conserved transcription factor motifs, chromatin accessibility, and promoter activity will be required to determine whether these sequence differences are associated with species-specific *SOX15* regulation.

Furthermore, multiple sequence alignment of *SOX15* amino acid sequences across different species showed that the similarity between porcine *SOX15* and mouse *Sox15* was 75.32%, 84.12% with human *SOX15*, and as high as 90% with *SOX15* from cattle and goats (Figure 1B). Collectively, these results confirm that *SOX15* is relatively conserved across species and may exert analogous regulatory functions.

During evolution, the regulation and function of the *SOX15* gene have undergone moderate divergence. The *SOX15* gene is hypothesized to have originated from an intron-containing ancestral gene in the *SOXB1* subgroup, which subsequently evolved into *SOX19a/b* in zebrafish, *SOXD* in Xenopus laevis, and *SOX15* in mammals. To clarify the evolutionary relationship of *SOX15* across species, we constructed a phylogenetic tree, which showed that *SOX15* exhibits similar evolutionary trajectories among mammals but is significantly divergent from that of arthropods such as fruit flies (Drosophila melanogaster) (Figure 1C).

### 3.2. Dynamic Expression of SOX15 in Porcine Early Embryos and Pluripotent Stem Cells

Subsequent analysis of *SOX15* expression across various stages of early embryonic development indicated that, within the GSE139512 dataset, *SOX15* expression began to upregulate at the 2-cell stage and peaked at the 8-cell stage (Figure 1D). Furthermore, analysis of the GSE112380 dataset demonstrated that *SOX15* expression remained high during the epiblast stage (Figure 1D). In contrast, analysis of *SOX15* expression across different embryonic stages in mice revealed that its expression also began to upregulate at the 2-cell stage and peaked at the 8-cell stage, while its expression was notably low during the epiblast stage (Figure 1E).

The specific expression pattern of *SOX15* during early embryonic development—characterized by its upregulation at these key stages—suggests that *SOX15* contributes to the maintenance of the dedifferentiated state of early porcine embryos. It is also suggested to be a potential contributor to the maintenance of stem cell pluripotency.

Therefore, investigating the function and regulatory mechanisms of *SOX15* in porcine embryo-derived stem cells is of great necessity.

### 3.3. Expression Analysis of SOX15 in Porcine ESCs with Different Pluripotency States

**Figure 2:**
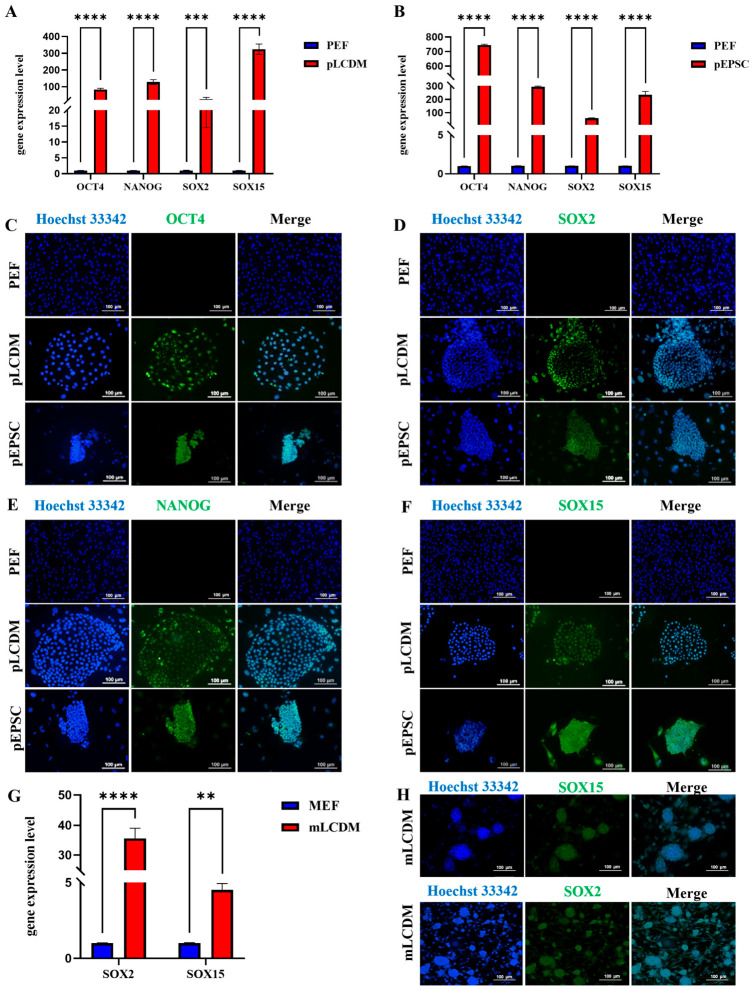
Dynamic expression of *SOX15* in porcine and mouse pluripotent stem cells. (**A**) Detection of core pluripotency factors (*OCT4*, *NANOG*, *SOX2*) and *SOX15* expression in pLCDM cells by qPCR; three biological replicates were performed; (**B**) detection of core pluripotency factors (*OCT4*, *NANOG*, *SOX2*) and *SOX15* expression in pEPSC cells by qPCR; three biological replicates were performed; (**C**–**E**) immunofluorescence analysis of pluripotency genes in pLCDM and pEPSC cells; (**F**) immunofluorescence detection of SOX15 expression in pLCDM and pEPSC cells; (**G**) detection of *Sox2* and *Sox15* expression in mLCDM cells by qPCR; three biological replicates were performed; (**H**) immunofluorescence analysis of Sox2 and Sox15 in mLCDM cells. * represents *p* < 0.05, ** represents *p* < 0.01, *** represents *p* < 0.001, and **** represents *p* < 0.0001.

To investigate the expression of *SOX15* in porcine embryonic stem cells, existing laboratory cell lines were selected for analysis. Porcine embryonic fibroblasts (PEF), which lack pluripotency, were used as the control group. The expression of pluripotency factors (*OCT4*, *SOX2*, *NANOG*) was first examined at both mRNA and protein levels in the cell lines (pLCDM, pEPSC). The results showed that the pEPSC and pLCDM cell lines exhibited high expression of core pluripotency factors (Figure 2A–F). Subsequent to rigorous validation of equivalent primer amplification efficiencies and stable internal reference gene normalization, quantitative PCR (qPCR) revealed that the transcript abundance of *SOX15* significantly exceeds that of *SOX2* in porcine embryo-derived stem cells.

To determine whether the expression pattern of *Sox15* in mouse embryonic stem cells resembles that in porcine embryonic stem cells, the laboratory-existing cell line mLCDM was used, and the expression of Sox2 and Sox15 was detected at both mRNA and protein levels. The results revealed that the expression of *Sox2* in mLCDM was significantly higher than that of *Sox15* (Figure 2G,H).

These findings suggest that *SOX15* is associated with the expression of pluripotency-related genes in porcine embryonic stem cells and may potentially contribute to the maintenance and transition of pluripotent states.

### 3.4. Antibody Validation and Knockdown System Construction

**Figure 3:**
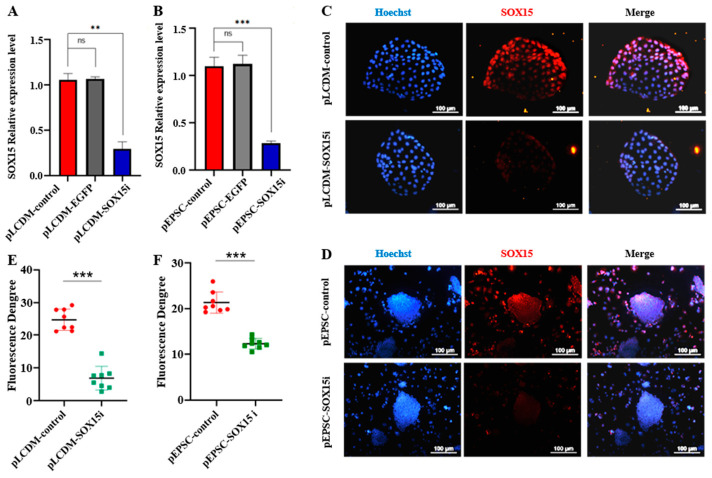
Analysis of *SOX15* knockdown efficiency. (**A**) *SOX15* expression in pLCDM-control, pLCDM-EGFP, and pLCDM-*SOX15*i cells, three biological replicates were performed; (**B**). *SOX15* expression in pEPSC-control, pEPSC-EGFP, and pEPSC-*SOX15*i cells; three biological replicates were performed; (**C**,**D**) SOX15 expression in pEPSC-control and pEPSC-SOX15i cells; (**E**,**F**) SOX15 expression in pLCDM-control and pLCDM-SOX15i cells (*n* = 3 independent biological replicates; 30 colonies were analyzed per replicate as technical subsampling). * represents *p* < 0.05, ** represents *p* < 0.01, and *** represents *p* < 0.001. ns: no significance.

Given the conservation of *SOX15* across species, an antibody with a recognition sequence similar to that of porcine *SOX15* was selected; however, its efficacy in porcine embryonic stem cells (pESCs) had not been validated. To confirm antibody specificity, an HA-tagged *SOX15* overexpression construct was generated (Supplementary Figure S2A). SOX15 and HA were co-stained in transfected 293T cells, revealing strong co-localization, indicating that the SOX15 antibody can specifically detect SOX15 in porcine embryonic stem cells (Supplementary Figure S2B). To investigate *SOX15* function in pESCs, a lentiviral miRNA-based knockdown system was established. A *SOX15*-specific miRNA sequence was designed using the RNAi Designer tool and cloned into the pcDNA™6.2-GW/EmGFP-miR vector, generating pcDNA™6.2-GW/EmGFP-*SOX15*miR. The miRNA cassette was subsequently inserted into the FUW-EGFP lentiviral backbone, yielding FUW-EGFP-*SOX15*miR, with FUW-EGFP serving as a control (Supplementary Figure S3A). Lentiviral packaging in 293T cells resulted in efficient viral production, and successful infection was confirmed by EGFP fluorescence (Supplementary Figure S3B). Two porcine embryonic stem cell lines, pLCDM and pEPSC, exhibiting different endogenous *SOX15* expression levels, were infected with the lentivirus. Infected cells displayed EGFP fluorescence 48 h post-infection (Supplementary Figure S4A,B), indicating stable viral integration. Knockdown efficiency was validated by qPCR, showing a 75% reduction in *SOX15* expression in pLCDM-*SOX15i* cells and 74% in pEPSC-*SOX15i* cells (Figure 3A,B), which was maintained across multiple passages (P3, P6, P9; Supplementary Figure S4C,D). Immunofluorescence analysis confirmed a corresponding decrease in SOX15 protein levels (Figure 3C–F). These results demonstrate that the lentiviral miRNA system enables stable and specific knockdown of *SOX15* in porcine embryonic stem cells.

### 3.5. SOX15 Knockdown Exhibits Impaired Self-Renewal- and Pluripotency-Associated Features of Porcine ESCs

**Figure 4:**
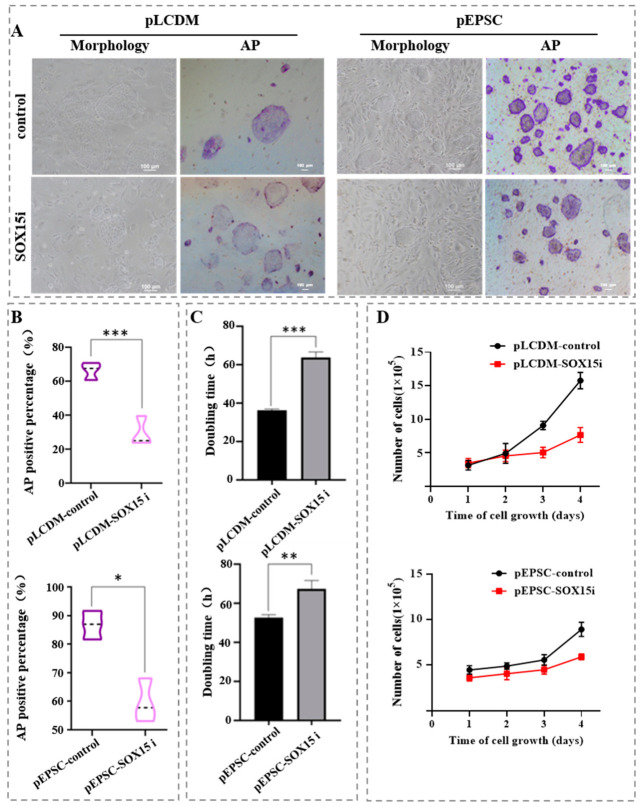
*SOX15* knockdown impairs colony morphology, alkaline phosphatase activity and proliferative capacity of porcine embryo-derived stem cells. (**A**) Effect of *SOX15* knockdown on morphology and AP staining in pLCDM and pEPSC cells; (**B**) AP staining positive rates in pLCDM and pEPSC cells with/without *SOX15* knockdown (*n* = 3 independent biological replicates; 50 colonies were analyzed per replicate as technical subsampling); (**C**) doubling time of pLCDM and pEPSC cells with/without *SOX15* knockdown (*n* = 3); (**D**) growth curves of pLCDM and pEPSC cells with/without *SOX15* knockdown (*n* = 3). * represents *p* < 0.05, ** represents *p* < 0.01, and *** represents *p* < 0.001.

**Figure 5:**
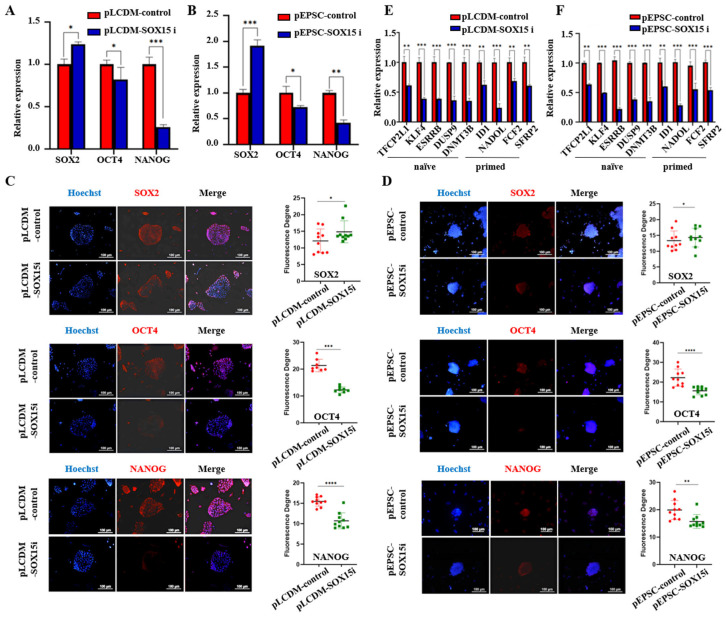
Knockdown of *SOX15* reduces the self-renewal and pluripotency of porcine embryonic stem cells. (**A**) qPCR analysis of pluripotency genes in pLCDM cells with/without *SOX15* knockdown. Three biological replicates were performed; (**B**) qPCR analysis of pluripotency genes in pEPSCs with/without *SOX15* knockdown. Three biological replicates were performed; (**C**,**D**) IF analysis of pluripotency factors in pLCDM and pEPSC cells with/without SOX15 knockdown; (**E**) qPCR analysis of Naïve and Primed markers in pLCDM cells with/without *SOX15* knockdown. Three biological replicates were performed; (**F**) qPCR analysis of Naïve and Primed markers in pEPSCs with/without *SOX15* knockdown. Three biological replicates were performed. * represents *p* < 0.05, ** represents *p* < 0.01, *** represents *p* < 0.001, and **** represents *p* < 0.0001. ns: no significance.

After knocking down *SOX15* in pLCDM and pEPSC cells, we observed that compared to the control groups, the *SOX15*-knockdown pLCDM cells exhibited slower growth, reduced clone numbers, smaller clones, and fewer alkaline phosphatase (AP)-positive cells. Similarly, *SOX15*-knockdown pEPSC cells also showed slower growth, weaker AP staining, and fewer AP-positive cells (Figure 4A,B). Statistical analysis revealed a significant difference in the AP-positive rate between the *SOX15*-knockdown cells and the control cells. The proliferation speed of pLCDM-*SOX15i* cells was similar to that of pLCDM-control cells from day 1 to day 2, but slowed down from day 2 to day 4. Similarly, the proliferation speed of pEPSC-*SOX15i* cells was consistent with that of pEPSC-control cells from day 1 to day 3, but slowed down from day 3 to day 4. Analysis of the cell doubling time showed a significant increase after *SOX15* knockdown (Figure 4C,D). These results indicate the impact of *SOX15* on cell morphology and proliferation capacity. We further detected the expression of pluripotency genes (*OCT4*, *SOX2*, *NANOG*) by qPCR. Since *OCT4*, *SOX2*, and *NANOG* are highly expressed in stem cells but expressed at very low levels in differentiated cells, detecting their expression levels after *SOX15* knockdown can preliminarily assess the pluripotent state of the cells. The results showed that in both pLCDM and pEPSC cells, *SOX2* expression increased after *SOX15* knockdown, while the expression of *OCT4* and *NANOG* decreased (Figure 5A,B). Detection at the protein level showed the same trend (Figure 5C,D). The expression of marker genes for Naïve and Primed pluripotency states was also detected by qPCR. We utilized established marker genes for validation. The results found that the expression of these pluripotency marker genes was reduced to varying degrees in the pLCDM-*SOX15i* group compared to the pLCDM-control group (Figure 5E). The expression of ESRRB, a marker gene for the Naïve state, was lower in pLCDM-*SOX15i* cells, and the expression of other pluripotency-related factors was also reduced to varying degrees compared to the pEPSC-control group (Figure 5F).

### 3.6. SOX15 Knockdown Leads to Abnormal Embryoid Body Formation and Imbalanced Trilayer Differentiation

**Figure 6:**
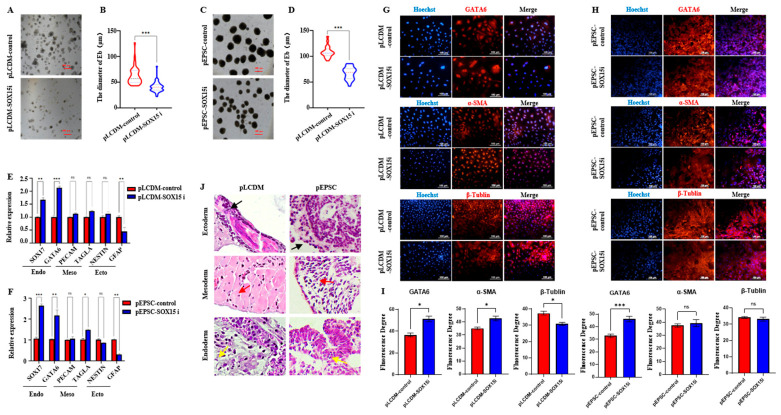
Knockdown of *SOX15* leads to abnormal formation of embryoid bodies (EBs) and imbalance in the differentiation of the three germ layers. (**A**) EB morphology of pLCDM cells with/without *SOX15* knockdown; (**B**) EB diameter analysis in pLCDM-control versus pLCDM-*SOX15*i cells (*n* = 3 independent biological replicates; 20 EBs were analyzed per replicate as technical subsampling.) (**C**) EB morphology of pEPSC cells with/without *SOX15* knockdown; (**D**) EB diameter analysis in pEPSC-control versus pEPSC-*SOX15*i cells (*n* = 3 independent biological replicates; 20 embryoid bodies were analyzed per replicate as technical subsampling); (**E**) qPCR analysis of germ layer markers in EBs from control and *SOX15*-knockdown pLCDM cells; three biological replicates were performed; (**F**) qPCR analysis of germ layer markers in EBs from control and *SOX15*-knockdown pEPSC cells; three biological replicates were performed; (**G**,**H**) IF analysis of trilineage markers in pLCDM and pEPSC cells with/without SOX15 knockdown; (**I**) SOX15 expression in pLCDM-control and pLCDM-SOX15i cells (*n* = 3 independent biological replicates; 30 colonies were analyzed per replicate as technical subsampling.); (**J**) Teratoma formation assay in vivo using pLCDM-control and pEPSC-control cells (Black arrows indicate ectoderm, red arrows indicate mesoderm and yellow arrows indicate endoderm). * represents *p* < 0.05, ** represents *p* < 0.01, and *** represents *p* < 0.001. ns: no significance.

The aforementioned results indicate that *SOX15* is a key factor in maintaining stem cell pluripotency. Consequently, the absence of *SOX15* appears to affect the subsequent differentiation potential of the cells. The spontaneous differentiation ability of cells after *SOX15* knockdown was examined. The culture medium for pLCDM and pEPSC cells was replaced with 15% FBS medium for suspension culture. On day 5 of culture in 15% FBS medium, the cells differentiated and formed embryoid bodies (EBs). Morphological observation revealed that, compared to the control groups, the pLCDM-*SOX15i* and pEPSC-*SOX15i* cells formed fewer EBs. These EBs were lighter in color and had a significantly smaller diameter (Figure 6A–D), indicating that they exhibited impaired proliferation and abnormal differentiation. This suggests that *SOX15* affects the induced differentiation capacity of stem cells. The expression of marker genes for the three germ layers was analyzed by qPCR after in vitro induced differentiation. In differentiated pLCDM-*SOX15i* cells, the expression of endoderm marker genes *SOX17* and *GATA6* was elevated. Similarly, differentiated pEPSC-*SOX15i* cells also showed high expression of endoderm markers. The expression of mesoderm marker genes *PECAM* and *TAGLN* showed no significant changes. In contrast, the expression of the ectoderm marker gene *GFAP* was reduced in both cell lines. As *GFAP* is a marker for neuronal generation, these results indicate that while *SOX15*-knockdown cells retain differentiation capability, their capacity to form well-structured embryoid bodies is severely compromised, and the uncoordinated differentiation into the three germ layers reflects a destabilized pluripotent network (Figure 6E,F). The EBs were transferred onto gelatin-coated plates and allowed to undergo spontaneous differentiation in 15% FBS medium for 7 days. After this period, the expression of the three germ layer markers α-SMA (mesoderm), β-tubulin (ectoderm), and GATA6 (endoderm) was detected (Figure 6G–I). The in vivo teratoma formation assay is the gold standard for evaluating the pluripotency and differentiation potential of stem cells. To assess differentiation capacity, cells were injected subcutaneously into immunodeficient mice. Specifically, 1 × 10^7^ cells from the pLCDM control group, pLCDM-*SOX15* knockdown group, pEPSC control group, and pEPSC-*SOX15* knockdown group were each subcutaneously injected into the neck region of approximately 7-week-old nude mice (with three mice per group). Teratoma formation was monitored over a period of 4 to 5 weeks. The results showed that all mice injected with pLCDM control and pEPSC control cells developed teratomas at the injection sites, with the tumor sizes ranging from 5 to 7 mm. In contrast, no teratoma formation was observed in any of the mice injected with cells from the *SOX15* knockdown groups. Hematoxylin and eosin (HE) staining of the excised teratomas revealed histological tissue-like structures consistent with derivatives of the three germ layers (Figure 6J). In summary, *SOX15* knockdown compromised embryoid body (EB) formation, teratoma formation, and the differentiation potential into the three germ layers, as evidenced by altered expression of germ layer-specific marker genes and spontaneous differentiation. Notably, the failure to form teratomas may reflect not only impaired differentiation capacity but also defects in cell survival or proliferation following transplantation.

## 4. Discussion

*SOX15* has long been regarded as a secondary or partially redundant member of the SOX family in mammalian pluripotency regulation, a perception largely stemming from mouse models where its deficiency yields minimal developmental phenotypes [12,14]. Conversely, our findings reveal expression patterns suggesting species-specific functions in pigs, thereby offering a complementary perspective to this rodent-centric interpretation.

Despite sharing a highly conserved HMG-box domain with *SOX2* and the capacity to bind *OCT4* for regulating SOX-OCT composite motifs [4], functional redundancy between these factors appears species-dependent. Unlike in mice—where *Sox2* dominates the pluripotency network, *Sox15* expression is low during the epiblast stage, and its deletion is tolerated—our data demonstrate that porcine *SOX15* is robustly upregulated from the 2-cell stage onward and remains highly expressed during the epiblast stage, coinciding with critical windows of pluripotency establishment and maintenance.

This distinct spatiotemporal pattern strongly implies evolutionary divergence in pluripotency regulatory hierarchies between rodents and large mammals. Supporting this, evolutionary sequence analysis indicates greater conservation of *SOX15* among large mammals than between pigs and mice, particularly in promoter regions, suggesting divergence in transcriptional regulatory mechanisms that may underlie its functional specialization in porcine embryogenesis.

Functionally, stable *SOX15* knockdown in porcine embryo-derived stem cells altered colony morphology, reduced alkaline phosphatase (AP) activity, slowed proliferation, and dysregulated core pluripotency genes. While altered colony morphology and reduced AP activity might reflect general declines in stem cell quality, the loss of teratoma-forming capacity and the downregulation of *OCT4* and *NANOG* indicate that *SOX15* depletion is associated with impaired pluripotency-associated features. However, because *SOX15* knockdown also reduced proliferation and prolonged cell doubling time, the current data cannot exclude the possibility that impaired cellular fitness, stress responses, or reduced viability contribute to these phenotypes. Therefore, our findings should be interpreted as evidence that *SOX15* supports pluripotency-associated cellular states in porcine embryo-derived stem cells, rather than as definitive proof that *SOX15* directly maintains the pluripotency network. Accordingly, additional studies, including global transcriptomic profiling, pluripotency scoring, directed differentiation assays, and mechanistic analyses, will be required to distinguish primary effects on pluripotency from secondary consequences of impaired cell fitness. Importantly, the concurrent increase in *SOX2* expression following *SOX15* depletion suggests a compensatory attempt; however, the failure to maintain *OCT4* and *NANOG* indicates this compensation is insufficient. This suggests that *SOX15* occupies a non-redundant nodal position within the porcine pluripotency network, distinct from the functional redundancy often observed in murine systems. This dynamic indicates that *SOX15* is not merely an isolated backup factor but an actively integrated component, potentially functioning as a modulator of cell state equilibrium rather than solely a direct regulator [17]. Alternatively, this may reflect a complex regulatory feedback loop integrating external signaling with core transcription factors [5,16].

Moreover, *SOX15* depletion compromises EB and teratoma formation and alters lineage specification, enhancing endodermal while reducing ectodermal potency. These findings suggest that *SOX15* contributes to sustaining pluripotency and may participate in modulating lineage specification in pigs. Unlike observations in murine models, this apparent lineage bias hints at a potential species-specific functional relevance of *SOX15* in porcine embryogenesis.

From a comparative biology perspective, our study highlights an important principle: core components of pluripotency networks may not be universally conserved in their functional hierarchy across mammals. While transcription factor families are evolutionarily conserved, their relative importance and network positioning can diverge significantly among species.

However, several limitations of this study should be acknowledged. First, although our comparative transcriptomic analyses revealed divergent *SOX15* expression patterns between pigs and mice, direct cross-species functional validation is still lacking. Therefore, the classification of *SOX15* as a lineage-specialized regulator remains correlative. Future cross-species rescue or knock-in experiments, such as replacing the murine *Sox15* locus with its porcine ortholog, will be required to establish a causal link between *SOX15* sequence evolution and functional divergence.

A second key caveat to our conclusions is the reliance on a single miRNAi construct for *SOX15* knockdown. Although consistent phenotypic changes were observed in two independent porcine pluripotent stem cell models, pLCDM and pEPSC, and knockdown efficiency was validated at both mRNA and protein levels, future studies using independent knockdown sequences, CRISPR interference, or RNAi-resistant *SOX15* rescue experiments will be necessary to more rigorously confirm that the observed phenotypes are specifically attributable to *SOX15* depletion rather than off-target effects.

A third critical limitation pertains to the consistency of experimental controls. While an FUW-EGFP empty vector control was employed during initial knockdown validation and showed no overt effect on *SOX15* expression, this control—along with a non-targeting miRNA control—was not incorporated into every downstream functional assay (e.g., AP staining, proliferation curves, EB formation, teratoma assays). Consequently, we cannot fully exclude the possibility that nonspecific effects arising from lentiviral transduction, GFP expression, random vector integration, or the miRNA cassette itself may have contributed to some of the observed phenotypes. This caveat is explicitly noted alongside the relevant results in the preceding sections. Future studies should integrate non-targeting miRNA and empty-vector controls across all functional readouts to definitively deconvolute *SOX15*-specific effects from those of the delivery system.

A fourth key limitation concerns the interpretation of our qPCR datasets. The qPCR marker panels in this study were employed for exploratory profiling of transcriptional trends rather than for statistical confirmation. Consequently, no correction for multiple testing was applied to these datasets; reported p-values serve a descriptive purpose to highlight fold-changes. Crucially, the core conclusions of this study are primarily substantiated by robust functional assays—including proliferation kinetics, alkaline phosphatase activity, and embryoid body formation—and by protein-level validation via immunofluorescence.

Finally, this study did not identify direct *SOX15* targets, binding sites, or interacting partners. Therefore, the findings should be interpreted as phenotypic rather than mechanistic. Future ChIP-seq, proteomic, and transcriptomic analyses will be required to define the *SOX15* regulatory network.

These findings have broader implications. First, they emphasize the necessity of studying pluripotency mechanisms directly in target species rather than extrapolating conclusions from rodent models. Second, identifying species-specific regulators such as *SOX15* may facilitate the optimization of culture systems and the establishment of stable naïve pluripotent stem cell lines in pigs. Finally, understanding how pluripotency networks evolve across mammals provides valuable insights into developmental plasticity, evolutionary adaptation, and the molecular logic underlying early embryogenesis.

The striking contrast between the severe impairment of porcine pluripotency upon *SOX15* depletion and the subtle phenotypes observed in *Sox15*-null mice underscores a potential evolutionary shift in the hierarchical organization of the pluripotency network. However, we acknowledge that a balanced, side-by-side functional assessment—such as comparing the effects of *Sox15* knockdown in mouse ESCs with our findings in porcine ESCs—is required to definitively attribute these differences to intrinsic functional specialization rather than species-specific compensatory mechanisms. Future reciprocal complementation assays, wherein porcine *SOX15* is expressed in murine *Sox15*-deficient backgrounds (or vice versa), will be instrumental in resolving this question and establishing definitive causal links between sequence evolution and functional divergence.

## 5. Conclusions

In summary, this study highlights *SOX15* as more than a presumed auxiliary factor, positioning it as a significant contributor to the robustness of the pluripotency program in pigs. By integrating evolutionary analysis, developmental transcriptomics, and functional perturbation experiments, we demonstrate that *SOX15* is associated with the maintenance of self-renewal, preserving colony integrity, sustaining the robust cellular fitness necessary for teratoma formation, and coordinating lineage potential in porcine embryo-derived stem cells. We note that definitive confirmation of *SOX15*-specific causality awaits future validation via independent knockdown sequences, CRISPR interference, or RNAi-resistant rescue experiments. These findings support the notion that lineage-specific adaptations can alter the functional contribution of individual regulatory components. In this context, *SOX15* appears to be a lineage-specialized factor that has acquired substantial regulatory influence in large mammals. While our data establish its functional indispensability, the precise molecular mechanisms such as specific genomic occupancy and protein interactors—require further investigation to fully define its role as a lineage-specific regulator. Beyond redefining the biological significance of *SOX15*, this work emphasizes the necessity of studying pluripotency directly within target species. Such comparative approaches are essential for establishing robust pluripotent stem cell systems in livestock species and for constructing a more evolutionarily informed framework of mammalian developmental biology.

## Data Availability

All data associated with this study are included in the main text and Supplementary Materials. Raw and processed quantitative data for all functional assays—including qPCR, quantitative immunofluorescence, alkaline phosphatase staining quantification, embryoid body diameter measurement, and proliferation assays—as well as publicly available transcriptomic analyses, processed expression matrices, and analysis scripts, have been provided in the Supplementary Materials and attachments.

## Supplementary Materials

The following supporting information can be downloaded at: https://www.mdpi.com/article/10.3390/cells15141283/s1, Supplementary Figure S1: Sequence analysis of SOX15. Supplementary Figure S2: Construction and Antibody Validation of HA-Tagged Vectors. Supplementary Figure S3: Development of the SOX15 knockdown system. Supplementary Figure S4: Efficient Generation of SOX15 Knockdown Cell Lines in Porcine LCDM and EPSC Cells. Table S1: Oligonucleotide sequences used in this study. Table S2: PCR primer sequences; mSOX15 is the mouse-derived SOX15 gene. Table S3: Statistics of AP-positive rates. Table S4: Measurements of EB diameters. Table S5: Statistics of proliferation curve data for pLCDM and pLCDM-SOX15i cells. Table S6: Statistics of proliferation curve data for pEPSC and pEPSC-SOX15i cells. Table S7: Quantification of Gene Expression by qPCR in PEF and pLCDM Cells. Table S8: Quantification of Gene Expression by qPCR in PEF and pEPSC.
