# Supplementary material for: SOX15 Contributes to the Maintenance of Pluripotency in Porcine Embryonic Stem Cells
Source: Cells. 2026 Jul 17;15(14):1283. doi: 10.3390/cells15141283 (PMC13406406; doi:10.3390/cells15141283)
Supplement: Supplementary file 1 [file cells-15-01283-s001.zip › supplementary.pdf]

SOX15 Contributes to the Maintenance of Pluripotency in Porcine Embryonic Stem Cells

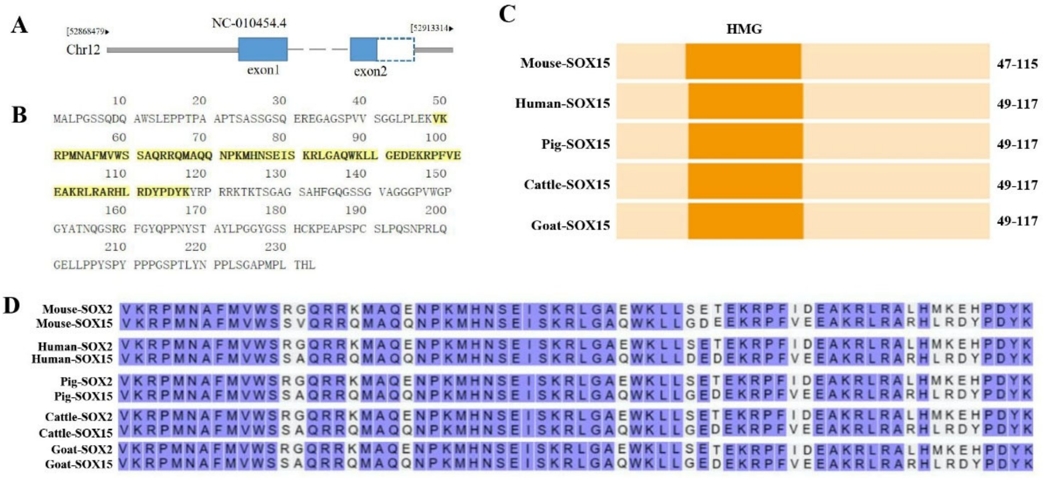

Figure S1. Sequence analysis of *SOX15*.

A, Locus information of porcine *SOX15*. B, Amino acid sequence of pig *SOX15*. C, HMG sequence alignment between different species of *SOX2* and *SOX15*. D, Location of amino acid sequence in the HMG region of the *SOX15* gene in rats, humans, pigs, cattle, and goats.

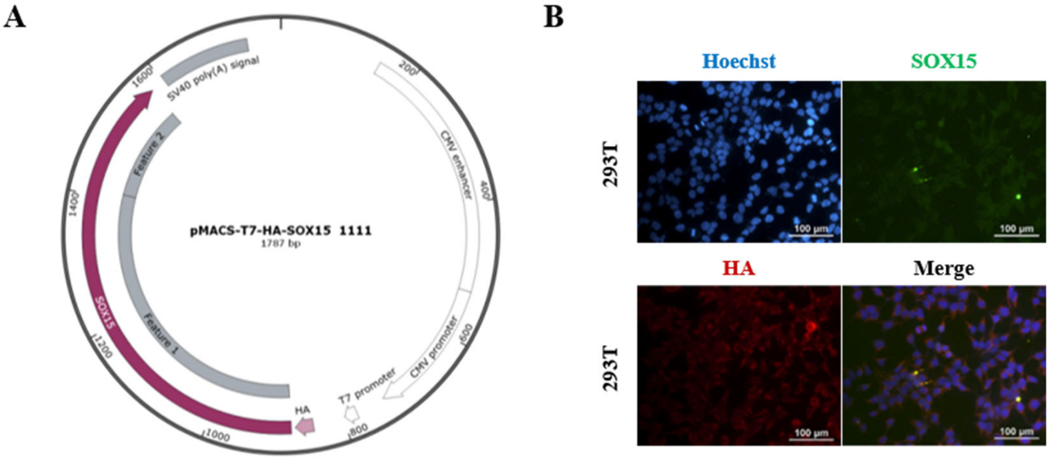

Figure S2. Construction and Antibody Validation of HA-Tagged Vectors.

A, pMACS vector carrying HA tag overexpression *SOX15*. B, Immunofluorescence detection of the expression of HA and SOX15.

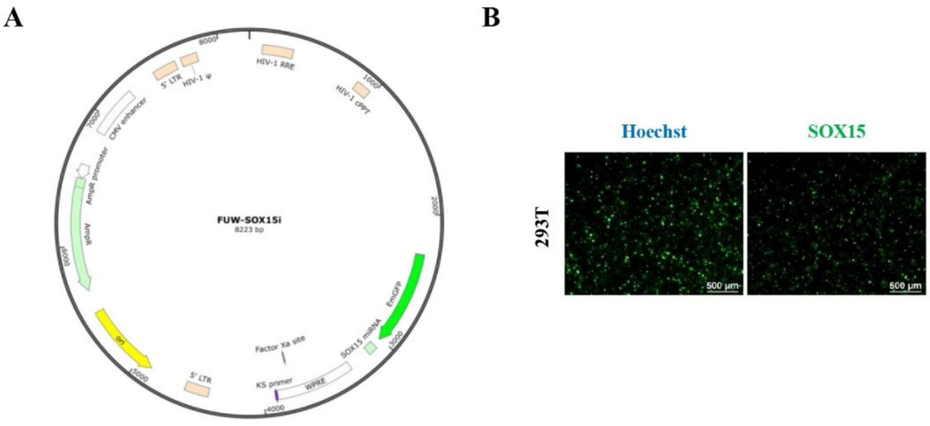

Figure S3. Development of the *SOX15* knockdown system.

A, pcDNATM6.2-GW/EmGFP-*SOX15*miR plasmid map. B, Green fluorescence expression of 293T cell packaging virus.

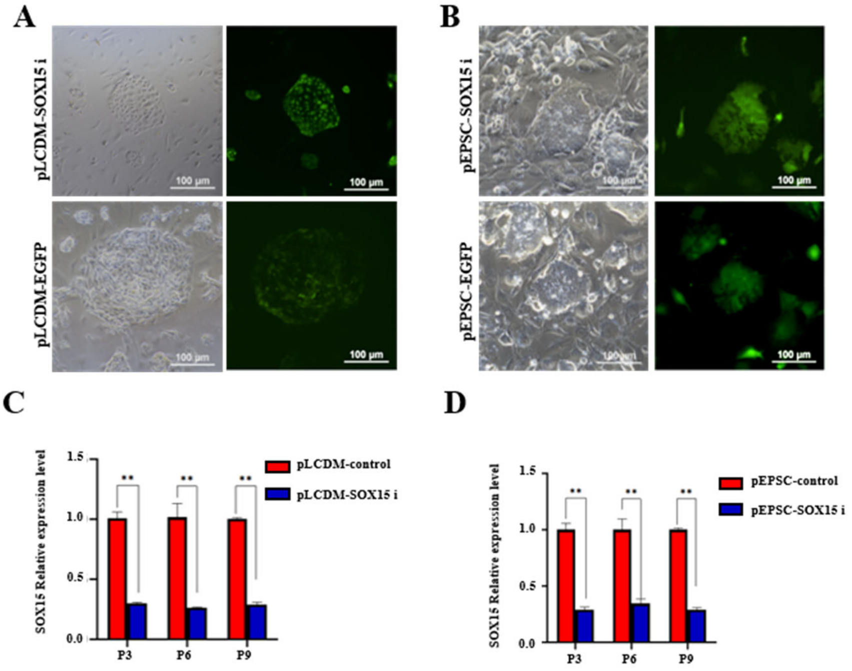

Figure S4. Efficient Generation of SOX15 Knockdown Cell Lines in Porcine LCDM and EPSC Cells

A, Brightfield and fluorescence imaging of pLCDM cells following lentiviral infection (pLCDM-control vs. pLCDM-SOX15i). B, Brightfield and fluorescence imaging of pEPSC cells following lentiviral infection (pEPSC-control vs. pEPSC-SOX15i).C, Relative *SOX15* mRNA expression levels in pLCDM cells at passages 3, 6, and 9. D, Relative *SOX15* mRNA expression levels in pEPSC cells at passages 3, 6, and 9.

Table S1: Oligonucleotide sequences used in this study

Name	Sequence (5'→3')	Notes
SOX15-F	CTACCGGTATGGCGCTACCCGGCTCCT	AgeI site
SOX15-R	CCGAATTCTTAGAGGTGGGTTAGAGGCATAGGG	EcoRI site
SOX15-miR-Osi-F	GATCTGCTGTTTACAGTGGGAAGAGCCGTAGTTTTGGCCACTGACTGACTACGGCTCCCCACTGTAAACAGG	RNAi oligo
SOX15-miR-Osi-R	CTAGACGACAAATGTCACCCTTCTCGGCATCAAAACCGGTGACTGACTGATGCCGAGGGGTGACATTTGTCC	RNAi oligo
miR-cassette-F	GTCTAGAATGGTGAGCAAGGGCGAG	XbaI site
miR-cassette-R	GGAATTCCTAGATATCTCGAGTGCGGCC	EcoRI site

Table S2: PCR primer sequences

GENE	Forward primer (5' -3')	Reverse primer (3' -5')
GAPDH	GCAAAGTGGACATTGTCGCCATCA	TCCTGGAAGATGGTGATGGCCTTT
NANOG	CCTCCATGGATCTGCTTATTC	CATCTGCTGGAGGCTGAGGT
OCT4	GAAGGTGTTCAGCCAAACGAC	CGATACTTGTCCGCTTTC
SOX2	AACCAGAAGAACAGCCCAGAC	TCCGACAAAAGTTTCCACTCG
KLF4	CATGAGTTGGGGGAGGGAAG	ACTCACCAAGCACCATCGTT
ESRRB	ACAAACTCTTCCTGGAGATGC	GGACTTGAATGTTGAGGCTG
NODAL	AACCAACCACGCATACATCCA	AGACACCCACATTCTTCCACAA
TFCP2L1	GCACTACAACCAGCACAACT	ATGGGCAGAGCAAGGAC
SOX15	CAGATGGCGCAGCAGAACCCGAAGA	CGCTTAGCCTCCTCCACGAA
NCAM	CCACCGTCACCGCTAACTC	CCAGCACGCTAGAGGACAAA
GATA6	TTGGTTATTCCCGAATTTCTCCG	CATTCCTGCAAACTGGGTGATAC
CDX2	GCTATAAATGCCAGAGCCAACC	AACAACCCAAACAGCAGCAAC
TFCP2L1	GCACTACAACCAGCACAACT	ATGGGCAGAGCAAGGAC
ID1	TTCTCACTCACCAGCCACCA	AAATAAAACAAAACACCCCACAG
TAGLN	CCATAAGAGGGAATTCACAGAGAG	CTGGGATGAGGAGACAGTAGAGC
SOX17	GACATGAAGATGAAGGGCGA	GTACTTGTAGTTGGGGTGGTCC
FGF2	GCGACCCTCACATCAAACT	CAGTGCCACATACCAACT

Table S3：Statistics of AP-positive rates.

	Replicate	AP positive rates (%)	Number of positive clones
pLCDM-control	1	72	36/50
	2	70	35/50
	3	62	31/50
pLCDM-SOX15i	1	22	11/50
	2	24	12/50
	3	38	19/50
pEPSC-control	1	82	41/50
	2	88	44/50
	3	92	46/50
pEPSC-SOX15i	1	52	26/50
	2	58	29/50
	3	68	34/50

Table S4: Measurements of EB diameters.

pLCDM-control	pLCDM-SOX15i	pEPSC-control	pEPSC-SOX15i
79.9	45.4	110.3	83.3
75.2	45.7	104.1	54.7
41.5	27.6	111.0	54.6
61.8	31.8	105.1	61.6
79.0	34.7	128.9	43.4
124.6	41.1	103.4	84.0
76.2	47.3	137.6	57.7
42.7	36.1	106.7	42.3
67.4	47.8	106.3	83.8
54.5	42.6	100.9	54.8
52.2	35.6	110.2	56.8
53.4	50.2	138.9	93.1
50.2	27.0	108.4	56.6
95.2	32.7	132.2	79.5
79.2	49.2	106.7	70.9
51.2	38.4	107.7	59.1
82.4	44.4	106.9	71.8
82.9	37.6	114.7	58.8
80.1	43.0	135.7	53.2
56.3	31.6	106.8	89.7
49.7	30.7	103.0	53.9
73.7	37.3	114.8	49.5
55.3	35.9	110.0	69.0
46.1	41.7	128.4	88.6
54.9	58.6	129.3	55.1
49.4	35.70	109.1	79.4
57.6	40.7	111.2	78.4
56.0	62.0	111.3	65.5
77.3	35.8	113.7	83.0
116.2	25.0	109.2	88.1
62.5	38.5	107.0	58.8
97.2	46.8	132.0	82.9
79.3	40.4	97.5	81.7

70.0	26.9	133.3	67.7
53.7	25.6	135.6	67.5
47.6	67.2	98.1	80.8
70.7	36.7	131.1	51.8
44.5	37.3	105.7	56.0
56.9	36.7	104.6	84.9
49.4	66.0	106.9	88.9
69.1	44.9	108.4	81.3
84.3	25.0	103.8	69.6
50.2	41.8	112.7	77.8
52.9	39.2	112.0	57.8
49.6	41.5	112.0	82.8
118.2	61.0	133.2	90.7
51.6	36.9	106.7	62.9
116.9	36.5	104.0	58.3
71.7	50.0	108.8	85.0
51.6	48.4	112.6	81.4
44.5	37.7	112.2	62.3
66.2	41.4	107.6	52.1
46.9	31.5	106.9	92.8
50.2	77.7	106.1	57.8
119.5	48.3	108.5	66.1
62.1	38.9	95.2	54.9
101.0	46.2	108.0	70.4
118.1	35.8	108.3	85.5
51.6	34.0	112.1	80.8
64.1	51.0	109.6	67.3

Table S5: Statistics of proliferation curve data for pLCDM and pLCDM-SOX15i cells.

Day	pLCDM-control_R1	pLCDM-control_R2	pLCDM-control_R3	pLCDM-SOX15i_R1	pLCDM-SOX15i_R2	pLCDM-SOX15i_R3
1	247235	311711	387351	280671	347181	418591
2	330778	517829	626643	373205	451002	544366
3	848484	906676	972031	423083	514763	580129
4	1443257	1608591	1681027	646164	795450	859986

Table S6：Statistics of proliferation curve data for pEPSC and pEPSC-SOX15i cells.

Day	pEPSC-control_R1	pEPSC-control_R2	pEPSC-control_R3	pEPSC-SOX15i_R1	pEPSC-SOX15i_R2	pEPSC-SOX15i_R3
1	404293	439622	496409	328692	353308	399237
2	451886	493381	521579	337236	410919	470234
3	497495	559443	613308	405648	441201	502706
4	843352	851856	981201	562282	582989	627181

Table S7: Quantification of Gene Expression by qPCR in PEF and pLCDM Cells.

	PEF	PEF	PEF	pLCDM	pLCDM	pLCDM
OCT4	0.988929	0.984108	1.027524	72.52109	89.09782	83.87211
NANOG	1.017344	0.994209	0.988678	135.99254	110.02003	134.44161
SOX2	0.911128	1.085529	1.011065	37.11793	18.38517	19.53719
SOX15	0.988679	1.017458	0.997862	352.74562	290.71541	328.15834

Table S8: Quantification of Gene Expression by qPCR in PEF and pEPSC.

	PEF	PEF	PEF	pEPSC	pEPSC	pEPSC
OCT4	0.95618	1.04261	0.99874	738.36845	749.65982	745.69812
NANOG	1.00980	0.98980	1.00940	298.23687	295.36975	286.46978
SOX2	0.99017	1.00621	1.00362	59.87934	56.76159	60.78934
SOX15	0.96841	1.01324	0.93185	230.95612	260.98345	210.96146

Supplementary Materials and Methods

Quantitative real-time PCR

All quantitative real-time PCR (qPCR) experiments were performed in accordance with the MIQE guidelines. Total RNA was extracted from cells using an RNA extraction kit (Invitrogen) according to the manufacturer's instructions. RNA concentration and purity were assessed by measuring absorbance at 260 and 280 nm. Genomic DNA contamination was removed by DNase treatment when necessary.

For reverse transcription, 1 μg of RNA was reverse-transcribed into cDNA using a reverse transcription kit (Abcam) according to the manufacturer's protocol. The same batch of reverse-transcribed cDNA was used for amplification to ensure a consistent normalization baseline for cross-gene comparison.

Each reaction contained cDNA template, gene-specific forward and reverse primers, qPCR Master Mix, and nuclease-free water in a final volume of 20 μL. The cycling conditions were as follows: initial denaturation at 95°C for 1 min, followed by 40 cycles of denaturation at 95°C for 30 s, annealing at 55°C for 30 s, and extension at 72°C for 30 s. Each sample was analyzed in technical triplicate.

GAPDH was used as the internal reference gene for normalization. Relative gene expression levels were calculated using the comparative Ct method, also known as the $2^{-\Delta\Delta Ct}$ method. For each sample, Ct values of the target genes were normalized to GAPDH to obtain ΔCt values, and relative expression levels were calculated relative to the corresponding control group.

Prior to formal sample analysis, primer amplification efficiencies were evaluated by generating standard curves using 5-fold serial dilutions of pooled cDNA templates. Standard curves were established for each primer set, and amplification efficiency was calculated based on the slope of each standard curve. The amplification efficiencies of all primer sets were above 95%, with standard curve R^2 values greater than 0.99, meeting the requirements for relative quantification.

Melt-curve analysis was performed at the end of each qPCR run to confirm amplification specificity. Each primer pair produced a single specific melt peak, with no evidence of primer-dimer formation or non-specific amplification. No-template controls were included to monitor possible contamination.

Cross-species transcriptome analysis of public datasets

Pig transcriptomic analysis included 72 individual single-cell RNA-seq libraries from two public datasets (GSE139512 and GSE112380). The analyzed stages comprised zygote (n = 3), 2-cell (n = 6), 4-cell (n = 13), 8-cell (n = 22), ICM (n = 3), and EPI (n = 25). For mouse, 12 low-input Smart-seq2 RNA-seq libraries from two public datasets (GSE71434 and GSE269651) were included. The analyzed stages comprised zygote (n = 2), 2-cell (n = 2), 4-cell (n = 2), 8-cell (n = 2), ICM (n = 2), and EPI (n = 2). Pig data were generated from individual single-cell RNA-seq libraries, whereas mouse data consisted of low-input Smart-seq2 RNA-seq libraries prepared from whole embryos or isolated embryonic tissues (ICM/EPI). All raw sequencing data were processed using a unified bioinformatics pipeline, including quality control, adapter trimming, read alignment to the same reference genome, gene-level quantification, and

normalization. No explicit batch effect correction was performed because the cross-species analysis was intended to compare overall developmental expression trends rather than absolute expression levels, and the results were interpreted as supportive rather than definitive evidence.
